# Niche Differentiation of Three Terrestrial Isopod Species Based on DNA Metabarcoding

**DOI:** 10.1002/ece3.71682

**Published:** 2025-06-26

**Authors:** Jiachen Wang, Yiwen Yang, Gaoji Zhang, Wei Xu, Hongyi Liu

**Affiliations:** ^1^ The Co‐Innovation Center for Sustainable Forestry in Southern China, College of Life Sciences Nanjing Forestry University Nanjing China; ^2^ Colleges of Forestry and Grassland Nanjing Forestry University Nanjing China

**Keywords:** dietary composition, DNA metabarcoding, isopods, trophic niche

## Abstract

Species coexistence is a hot topic in ecology, with niche differentiation playing a key role in reducing interspecific competition. This study investigates the dietary habits and niche differentiation of three terrestrial isopod species ( *Armadillidium vulgare* , *Sphaeroma raffaelei*, and *Trachelipus semiproiectus* ) using DNA metabarcoding technology. By analyzing plant food composition, we identified significant differences in dietary preferences and niche widths among the species, with niche width (B) values of 13.3987 for *T. semiproiectus* , 10.0396 for *S. raffaelei* , and 6.0363 for *A. vulgare* . *Trachelipus semiproiectus* exhibited the broadest trophic niche, consuming a diverse range of plant taxa, including Bryopsida and Polypodiopsida, while *A. vulgare* and *S. raffaelei* showed more specialized diets dominated by Sapindales and Rosales, respectively. Dietary overlap indices (*O*_*jk*_) revealed moderate overlap between *T. semiproiectus* and *A. vulgare* (0.48) and *S. raffaelei* (0.39), whereas *A. vulgare* and *S. raffaelei* showed the lowest overlap (0.29). The results highlight the importance of dietary specialization and resource partitioning in facilitating species coexistence. This study provides new insights into the feeding ecology of terrestrial isopods and contributes to understanding soil invertebrate community dynamics, offering a foundation for biodiversity conservation and ecosystem management.

## Graphical abstract

Species coexistence is a hot topic in ecology, with niche differentiation playing a key role in reducing interspecific competition. This study investigates the dietary habits and niche differentiation of three terrestrial isopod species ( *Armadillidium vulgare* , *Sphaeroma raffaelei*, and *Trachelipus semiproiectus* ) using DNA metabarcoding technology. This study provides new insights into the feeding ecology of terrestrial isopods and contributes to understanding soil invertebrate community dynamics, offering a foundation for biodiversity conservation and ecosystem management.

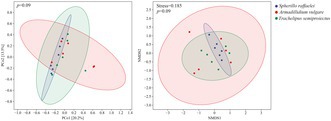

## 1 Introduction

Species coexistence is one of the key research focuses in ecology. Understanding the occurrence and maintenance of species coexistence is of great significance to the development of community ecology theory and is also a key focus in biodiversity research. Researchers have proposed various theories and hypotheses to explain coexistence mechanisms, including the resource‐ratio hypothesis (Huisman and Weissing 1999), the intermediate disturbance hypothesis (Grime 1973), and the niche differentiation theory (Udvardy 1959). Species with similar survival needs often exhibit niche differentiation (Udvardy 1959; Hardin 1960), and the outcome of competition leads to the differentiation of resources such as dietary composition, habitat, and time, thereby facilitating the coexistence of multiple species within a habitat. In terrestrial invertebrates like isopods, such niche differentiation may be mediated by functional traits such as mouthpart morphology or locomotor ability, which influence resource acquisition. Dietary analysis is a crucial component of nutritional niche research, reflecting animals' adaptation to their habitats based on their nutritional needs (Cooke and Crowley 2018). Furthermore, it is a crucial foundation for investigating fundamental ecological processes, including nutrient cycling in food webs, evaluating factors affecting species persistence, and analyzing interspecific interactions such as competition and predation (Marshal et al. 2008; Gong et al. 2017).

Given the importance of dietary analysis in understanding species coexistence, studying the dietary habits of soil invertebrates, such as terrestrial isopods, is particularly crucial, as they play significant roles in ecosystems. Invertebrates are integral to the trophic structure of soil food webs and essential for ecosystem functioning, yet they remain largely understudied and undervalued (Eisenhauer et al. 2019). Soil invertebrates, including nematodes, annelids, and arthropods, display exceptional diversity, representing up to 23% of global biodiversity and constituting 40%–80% of the animal biomass in soil ecosystems (Decaënsa et al. 2006; Fierer et al. 2009). Recent studies further underscore their crucial contributions to human well‐being by regulating a range of ecosystem processes (Chen et al. 2022). Among these, soil arthropods, particularly terrestrial isopods, exemplify the diverse ecological roles invertebrates play.

Terrestrial isopods are an important group within soil arthropods. They represent an ideal study group due to their high abundance in terrestrial ecosystems, pivotal role in nutrient cycling as decomposers, and physiological sensitivity to environmental changes, which together make them valuable indicators of soil health and ecosystem functionality. Terrestrial isopods are conventionally classified as detritivores, primarily feeding on decomposing plant material (Zimmer 2002). However, evidence suggests they also feed on fresh plant components, such as leaves and young seedlings, as well as animal dung and carrion (Zimmer and Topp 2002). *Armadillidium vulgare* is known to switch between feeding on dead and live plant material in the field (Saska 2008). Although some species show a low tendency to disperse and aggregate under stones, logs, or haystacks (Catalán et al. 2007), other species are very vagile when foraging for food. The three species targeted in this study, *A. vulgare* , *Sphaeroma raffaelei*, and *Trachelipus semiproiectus* , are ecologically significant components of the soil fauna in our study region. As key detritivores and potential herbivores, they play crucial roles in nutrient cycling (e.g., breaking down leaf litter, influencing decomposition rates), soil structure modification (through burrowing and fecal pellet production), and potentially seed dispersal. Despite their ecological importance and sympatric distribution in the study area, detailed knowledge of their specific feeding ecology and niche differentiation in natural settings remains scarce (Catalán et al. 2007). Research on isopods has predominantly focused on taxonomic aspects, while studies on the feeding habits of terrestrial isopods are relatively limited (Catalán et al. 2007). Clarifying their food composition and feeding strategies can help elucidate the mechanisms of coexistence among various terrestrial isopod species from the perspective of nutritional niche. It also provides a theoretical foundation for maintaining species diversity and managing the population ecology of terrestrial isopods.

However, traditional methods face limitations in accurately assessing the dietary composition of terrestrial isopods, necessitating more advanced techniques to overcome these challenges. Accurately and efficiently evaluating dietary composition through direct observation is challenging. Direct observational approaches include gut content microscopy and fecal fragment analysis. A key drawback of traditional observational approaches is that food items are frequently digested extensively, rendering the identification of their remnants difficult from a taxonomic perspective (Hubbell et al. 1965; Akrim et al. 2018). Particularly in situations of food scarcity, where foods vary, are small in size, and quickly break down in the digestive tract, direct identification becomes challenging because the chyme consists of a blend of deteriorated food fragments (Hubbell et al. 1965; Bessey et al. 2019). Stable isotope analysis, while useful for distinguishing broad dietary categories, lacks resolution at the species level and cannot identify specific plant taxa consumed (Bohmann et al. 2018). Currently, DNA metabarcoding technology holds immense potential in the field of animal dietary studies, significantly enhancing the precision of dietary analysis. Compared to traditional methods, DNA metabarcoding offers clear advantages: it is largely unaffected by food digestion, enables the identification of morphologically unrecognizable food, increases the accuracy of species identification without relying on taxonomic expertise, and allows for the simultaneous analysis of multiple samples. This technology has been widely applied in molecular dietary studies of arthropods (Ibanez et al. 2013; Kajtoch and Mazur 2015; Cheng and Lin 2016; Bortolin et al. 2018).

The study site selected for this study exhibits high species diversity, with three terrestrial isopod species overlapping in distribution within this region, providing ideal conditions for studying niche overlap and coexistence mechanisms. Based on DNA metabarcoding technology, this study identified and compared the plant food composition of three isopod species, calculated the interspecific food niche overlap index and food niche width, and explored the mechanism of coexistence of these isopods from the perspective of the nutritional niche.

## 2 Materials and Methods

### 2.1 Study Area and Sample Collection

**FIGURE 1:**
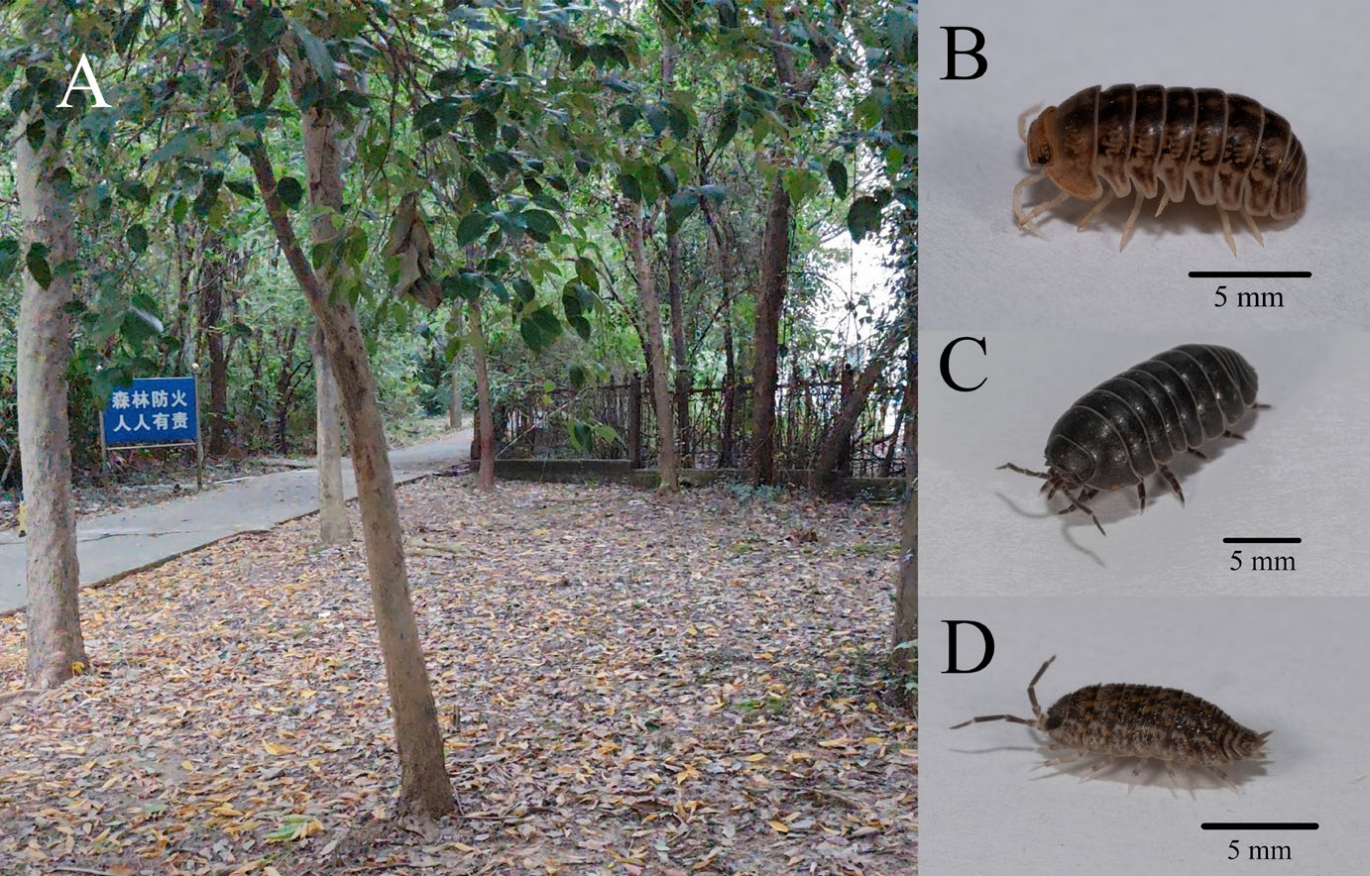
Study site and representative specimens of the three species. (A) The ecological setting of the sampling site. (B) The specimen of *Sphaeroma raffaelei*. (C) The specimen of *Armadillidium vulgare* . (D) The specimen of *Trachelipus semiproiectus*.

In September and October 2024, a total of 54 specimens were collected from eight dispersed sampling sites on the study site of Nanjing Forestry University (32.0777° N, 118.8165° E) (Figure 1), comprising 18 individual samples per species for *A. vulgare* , *S. raffaelei* , and *T. semiproiectus* . Active search with soft‐bristle brushes and forceps under decaying logs and leaf litter. Collected specimens were immediately transferred in groups of three to individual sterile Petri dishes. Moistened filter paper was used to maintain > 80% relative humidity. All specimens were kept under ambient laboratory conditions (25°C ± 1°C). Specimens were held for 6–12 h to allow gut clearance. Fecal pellets were collected using sterile micro‐spatulas. Each sample was flash‐frozen in liquid nitrogen within 30 s of collection. After feces were collected, the specimens were released back into the wild. The fecal samples were then transferred to small sterile tubes and stored at −80°C. This noninvasive approach avoids euthanasia or invasive procedures while yielding higher DNA integrity than gut contents, which suffer rapid degradation from host nucleases and pH extremes, whereas fecal DNA is stabilized by environmental exposure and bacterial encapsulation (Nichols et al. 2012; Deagle et al. 2019). This study was approved by the Laboratory Animal Welfare and Ethics Committee of Nanjing Forestry University ID:2023020.

### 2.2 DNA Extraction and Sequencing

Genomic DNA was extracted using the MagBeads Fast DNA Kit for Soil (MP Biomedicals, Santa Ana, USA) following the manufacturer's instructions. DNA quantity and quality were assessed with a NanoDrop NC2000 spectrophotometer (Thermo Fisher Scientific, Waltham, USA) and agarose gel electrophoresis, respectively. PCR amplification of the RuBisCO Large subunit region was performed using the forward primer rbcL‐F (5′‐ATGTCACCACCAACAGAGACTAAAGC‐3′) and the reverse primer rbcL‐R (5′‐CGTCCTTTGTAACGATCAAG‐3′). The rbcL primer, due to its high conservativeness and moderate mutation rate, is an ideal marker for studies on plant herbivory (Hofreiter et al. 2000; Dong et al. 2024). The PCR components contained 5 μL of buffer (5×), 0.25 μL of Q5 High‐Fidelity DNA Polymerase (5 U/μL) (New England Biolabs, Ipswich, USA), 1 μL of each primer (10 μM), 2 μL of dNTPs (2.5 mM), 1 μL of DNA template, and 14.75 μL of ddH_2_O. Thermal cycling consisted of initial denaturation at 98°C for 3 min, followed by 35 cycles consisting of denaturation at 98°C for 30 s, annealing at 50°C for 30 s, and extension at 72°C for 45 s, with a final extension of 5 min at 72°C. PCR amplicons were purified with Vazyme VAHTS DNA Clean Beads (Vazyme, Nanjing, China) and quantified using the Quant‐iT PicoGreen dsDNA Assay Kit (Invitrogen, Carlsbad, USA). After the individual quantification step, amplicons were pooled in equal amounts, and pair‐end 2 × 250 bp sequencing was performed using the NovaSeq 6000 platform (llumina, San Diego, USA) at Shanghai Personal Biotechnology Co. Ltd. (Shanghai, China).

### 2.3 Bioinformatics and Data Quality Filtering

Raw sequence data were demultiplexed using the demux plugin, and primers were trimmed with the cutadapt plugin (Martin 2011). Sequences were then merged, quality‐filtered, and dereplicated using fastq_mergepairs, fastq_filter, and derep_fullength in Vsearch. To optimize chimera removal and reduce computational complexity, unique sequences were first clustered at 98% similarity (cluster_size) followed by de novo chimera detection (uchime_denovo). This stringent initial clustering groups near‐identical sequences (e.g., PCR/sequencing errors), enhancing chimera detection efficiency. The resulting nonchimeric sequences were then re‐clustered at 97% similarity to define biologically relevant operational taxonomic units (OTUs), generating representative sequences and an OTU table. Nonsingleton OTUs were aligned with mafft (Katoh 2002) and a phylogeny was constructed using fasttree2 (Price et al. 2009). Taxonomy was assigned to OTUs using the classify‐sklearn naive Bayes classifier in the feature‐classifier plugin against the NCBI Database. NCBI provides the most comprehensive public repository for plant sequences (> 3.6 million plant entries as of 2024) (Kõljalg et al. 2013; Bokulich et al. 2018) (http://www.ncbi.nlm.nih.gov).

### 2.4 Statistical Analysis

The raw data files were submitted to Shanghai Personal Biotechnology Co. Ltd. (Shanghai, China) for processing and analysis. Sequence data were analyzed using QIIME2 and R packages (v3.2.0). OTU‐level alpha diversity indices, including Chao1, Observed species, Shannon, and Simpson indices, were calculated from the OTU table in QIIME2 and visualized as box plots. Ranked abundance curves were generated to compare OTU richness and evenness among samples. Beta diversity analysis was conducted using Jaccard, Bray–Curtis, and UniFrac metrics and visualized via PCoA and NMDS (Bray and Curtis 1957; Lozupone et al. 2007; Ramette 2007). PERMANOVA was used to assess microbiota structure differentiation among groups (McArdle and Anderson 2001). Taxonomy compositions and abundances were visualized using MEGAN and GraPhlAn (Asnicar et al. 2015). LEfSe was used to detect differentially abundant taxa (Segata et al. 2011), whereas OPLS‐DA was also applied. Random forest analysis was applied for group discrimination using QIIME2 (Breiman 2001). Based on the relative abundance of food composition at different taxonomic levels, this study used the Levins index (B) (Hurlbert 1978) to describe the food niche width and the Pianka overlap index (O_jk_) to measure the degree of food niche overlap (Pianka 1973).

The calculation formulas are as follows:

$$B=\sum P_{j}$$

where *B* represents the niche width and *P*_*j*_ represents the proportion of food item *j*.

$$O_{\mathrm{jk}}=\frac{\sum \left(p_{\mathrm{ij}}\cdot P_{\mathrm{ik}}\right)}{\sum P_{\mathrm{ij}}\cdot \sum P_{\mathrm{ik}}}$$

where *P*_*ij*_ and *P*_*ik*_ represent the relative abundance of food item *i* in the diets of species *j* and *k*, respectively. The range of *O*_*jk*_ is between 0 and 1. According to the criteria of Krebs (1999), an *O*_*jk*_ > 0.3 indicates a significant overlap, and *O*_*jk*_ > 0.6 indicates a highly significant overlap.

## 3 Result

### 3.1 Sequencing Results

A total of 18 rbcL barcode amplicons were successfully sequenced using the Illumina platform. The libraries generated 2,026,470 raw sequences based on rbcL amplification, with an average of 112,581 sequences per sample. After quality filtering, the number of sequences was reduced to 1,925,452, with an average of 107,970 sequences per sample. Rarefaction curves plateaued for all samples, confirming that sequencing depth was sufficient to capture dietary diversity. These sequences corresponded to 428 distinct OTUs, which were manually screened, and a total of 76 OTUs were identified.

While powerful, this approach has inherent limitations: Primer specificity (rbcL bias) may underrepresent certain plant taxa; PCR amplification biases could distort relative abundances; Incomplete reference databases (e.g., NCBI) limit species‐level resolution. These factors may affect dietary inference accuracy, particularly for quantitative assessments. We mitigated impacts by focusing on higher taxonomic ranks (order/genus) and using stringent bioinformatic controls. Despite constraints, metabarcoding remains superior for deciphering complex diets of cryptic invertebrates.

### 3.2 Dietary Composition

**FIGURE 2:**
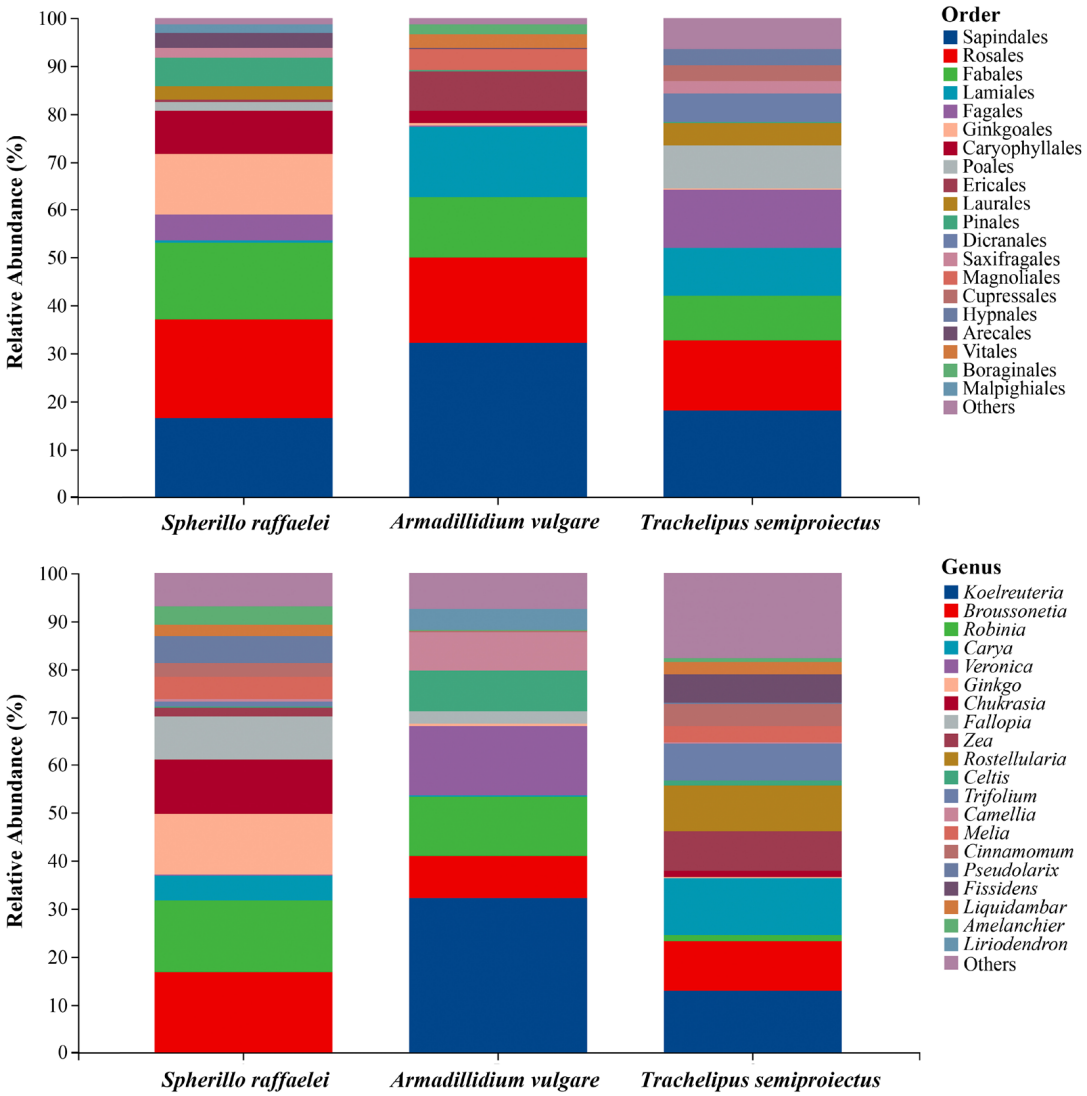
Relative abundance of plant taxa in the diet of each predator species.

The plant food items of three isopods represent 33 taxonomic orders, 47 families, 64 genera, and 65 identified species. At the class level, Bryopsida and Polypodiopsida in the dietary composition of *T. semiproiectus* are not consumed by the other two species. At the order level, 18 orders of identified taxonomic units had a relative abundance greater than 1%. Among them, Sapindales, Rosales, and Fabales constitute the primary plant food items for the three species, with these three orders together accounting for 52.54% of the plant food items (Figure 2). *Armadillidium vulgare* and *T. semiproiectus* have the highest proportion of Sapindales in their plant food items (32.35% and 18.15%, respectively). *Sphaeroma raffaelei* consumes slightly less Sapindales (16.51%) but shows a preference for Rosales (20.58%). At the genus level, *Broussonetia* is the most abundant plant food item in the diet of *S. raffaelei* (16.66%). *Armadillidium vulgare* and *T. semiproiectus* show a strong preference for *Koelreuteria* (32.22% and 12.92%), with *Broussonetia* also making up a significant proportion of their diet (8.67% and 10.27%) (Figure 2).

**FIGURE 3:**
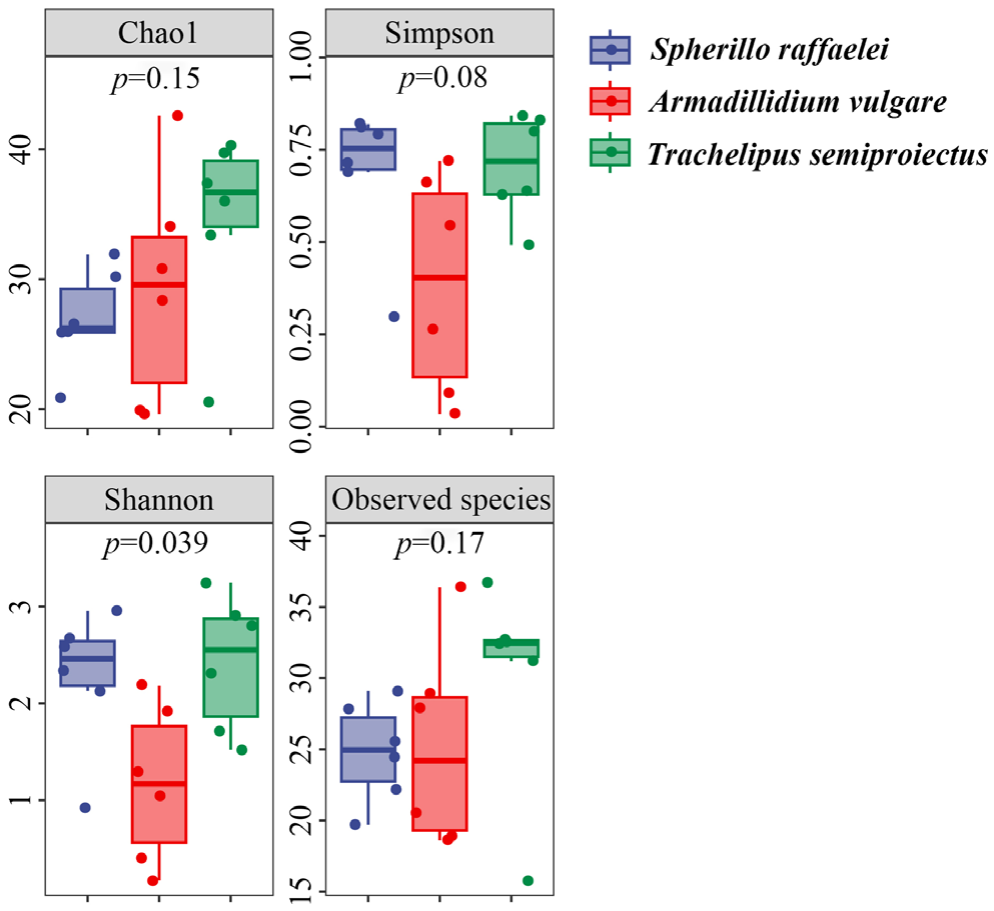
Dietary diversity (Chao 1, Simpson, Shannon, and observed species indexes) of each species.

**FIGURE 4:**
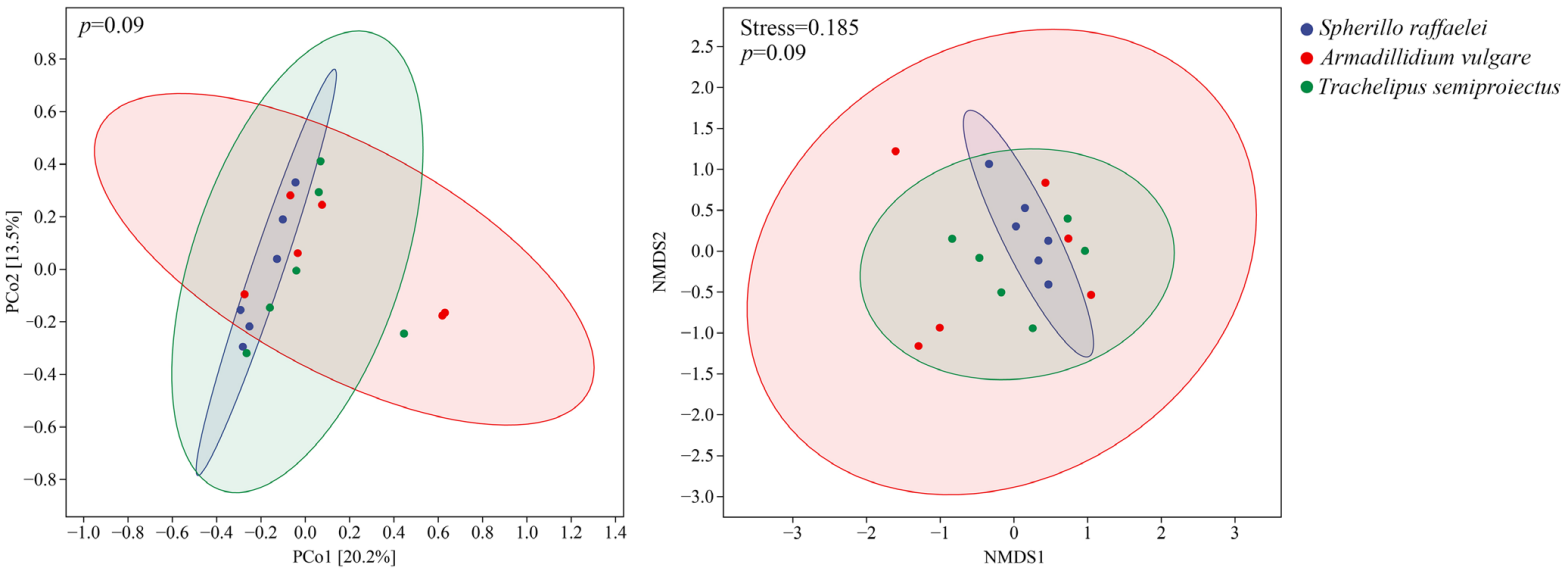
PCoA ordination plot and NMDS based on Bray–Curtis dissimilarity were used to visualize the differences in diet composition among samples. Each point corresponds to a single fecal sample, and ellipses represent 95% confidence intervals for each group.

The Chao1 and Observed species values of *T. semiproiectus* were higher than those of the other two species. The Simpson and Shannon indices of *A. vulgare* were lower than those of the other two species. Overall, most alpha diversity metrics showed no significant differences among the three isopod species (*p* > 0.05 for Chao1, Observed species, and Simpson indices) (Figure 3). However, the Shannon diversity index of *A. vulgare* was significantly lower than that of both *S. raffaelei* and *T. semiproiectus* (*p* = 0.039), indicating less even distribution of dietary components in this species. The NMDS and PCoA results based on Bray‐Curtis distances showed no significant differences in the plant food items among the three species (Figure 4). Statistical testing of beta diversity differences via PERMANOVA revealed no significant differences between any pair of groups. Specifically, the comparison between *S. raffaelei* and *A. vulgare* yielded a pseudo‐*F* = 1.64, *p* = 0.073; between *S. raffaelei* and *T. semiproiectus* : pseudo‐*F* = 1.21, *p* = 0.248; and between *A. vulgare* and *T. semiproiectus* : pseudo‐*F* = 1.21, *p* = 0.229. After adjusting for multiple comparisons using the *q*‐value method (*q* = 0.219, 0.248 and 0.248), all comparisons remained nonsignificant (*q* > 0.2). This lack of overall differentiation contrasts with the LEfSe‐identified taxa‐specific differences and niche width variations, suggesting that while certain plant taxa show species‐specific consumption patterns, the global dietary composition overlaps substantially at the community level.

**FIGURE 5:**
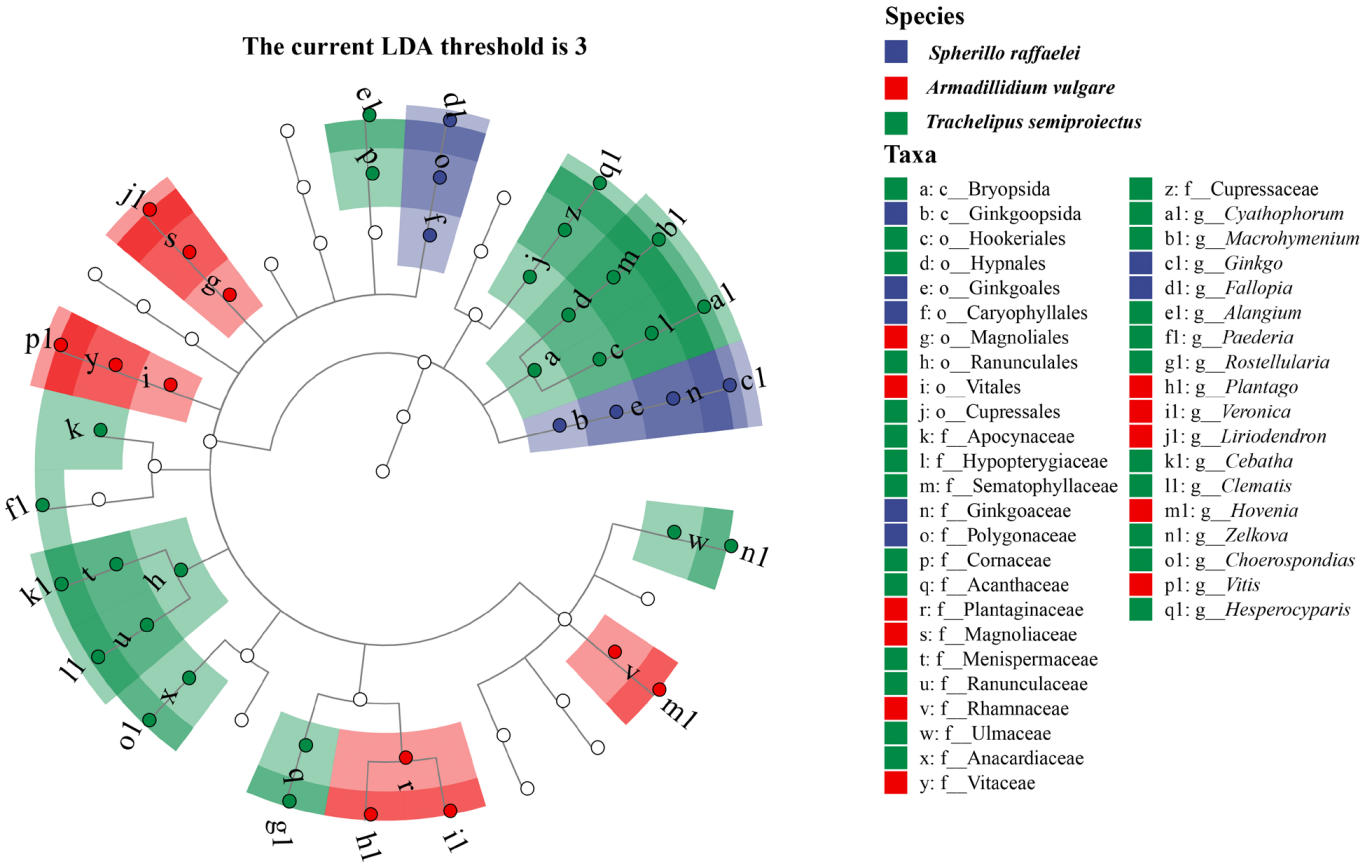
Cladogram generated from LEfSe analysis, illustrating differentially abundant taxa among experimental groups. The cladogram is structured with concentric circles radiating from the center, representing taxonomic levels from phylum to genus (or species). Each small circle at different taxonomic levels represents a taxon at that level. Coloring principle: Taxa with no significant differences are left uncolored, while differentially abundant taxa are colored according to their associated groups. Additionally, phylogenetic levels are indicated by the letters preceding the underscores (c = class, o = order, f = family, g = genus).

LEfSe was conducted to identify the key OTUs responsible for the differences in dietary item composition. The relatively high abundance of 18 OTUs (2 classes, 8 orders, 16 families) in specific hosts contributed to the observed differences. These OTUs were mainly found in *T. semiproiectus* , while *A. vulgare* had fewer OTUs, primarily from Plantaginaceae, Magnoliaceae, Rhamnaceae, and Vitaceae. Ginkgoaceae and Polygonaceae were predominantly found in the dietary items of *S. raffaelei* . The key OTUs at the genus level were *Cyathophorum*, *Macrohymenium*, *Ginkgo*, *Fallopia*, *Alangium*, *Paederia*, *Rostellularia*, *Plantago*, *Veronica*, *Liriodendron*, *Cebatha*, *Clematis*, *Hovenia*, *Zelkova*, *Choerospondias*, *Vitis*, and *Hesperocyparis* (Figure 5).

### 3.3 Niche Differentiation and Degree of Dietary Composition Overlap

Among the three isopods examined, the trophic niche width at the genus level revealed that *T. semiproiectus* from the family Trachelipodidae had the broadest niche for herbivorous food sources (*B* = 13.3987), followed by *S. raffaelei* (B = 10.0396) and *A. vulgare* (*B* = 6.0363), both from the family Armadillidiidae. Furthermore, the results for trophic niche width at the order and family levels closely aligned with those observed at the genus level (Table 1).

**TABLE 1:** Trophic niche breadth of *Armadillidium vulgare* , *Sphaeroma raffaelei*, and *Trachelipus semiproiectus* .

	Class level	Order level	Family level	Genus level
*Spherillo raffaelei*	1.4566	7.8052	8.7946	10.0396
*Armadillidium vulgare*	1.0134	5.4367	5.9964	6.0363
*Trachelipus semiproiectus*	1.3584	9.5398	12.6896	13.3987

The three isopods displayed varying degrees of dietary overlap. *Armadillidium vulgare* showed the highest overlap with *T. semiproiectus* (*O*_*jk*_ = 0.48), followed closely by *S. raffaelei* and *T. semiproiectus* (*O*_*jk*_ = 0.39), whereas the lowest overlap was observed between *A. vulgare* and *S. raffaelei* (*O*_*jk*_ = 0.29).

## 4 Discussion

The dynamics within a community are heavily influenced by how organisms interact with their environment, particularly through their niche parameters. Food, as a critical component of the niche, plays a significant role in defining an organism's ecological niche. By examining the dietary habits of animals, researchers can gain valuable insights into niche differentiation. Comparative analyses of diets among closely related species within the same community offer a direct approach to exploring this topic, revealing key aspects of autecology and the structure of food web interactions, as demonstrated in studies on arthropods (Furlong 2014; Bortolin et al. 2018).

Despite the importance of dietary studies in understanding niche differentiation, research on the feeding habits of terrestrial isopods in natural settings remains limited (Allen et al. 2023). Our study represents the first comparative investigation of the diet of different terrestrial isopod species in natural settings, addressing a significant gap in the existing literature. For this group, field‐based food preferences have rarely been reported, and only for very few species. Most observations were conducted in captivity, based on supplied food. Captivity‐based studies may oversimplify trophic interactions, as they cannot account for microhabitat variability, seasonal food availability, or intraspecific competition in natural ecosystems. While plant consumption under natural conditions remains largely unexplored (Catalán et al. 2007; Eberl 2012).

By employing DNA metabarcoding technology, while acknowledging inherent limitations such as detection biases and inability to quantify exact consumption, we were able to achieve high‐resolution identification of their plant food sources, revealing significant differences in dietary composition and niche width among the species. The broader niche width observed in *T. semiproiectus* (*B* = 13.3987) compared to *A. vulgare* (*B* = 6.0363) and *S. raffaelei* (*B* = 10.0396) suggests that *T. semiproiectus* utilizes a more diverse range of plant resources, potentially reducing direct competition with the other two species. These differences in niche breadth reflect distinct ecological strategies: *T. semiproiectus* 's generalist feeding reduces competition pressure by utilizing diverse resources, while *A. vulgare* 's specialization enhances efficiency in exploiting specific niches. This partitioning promotes coexistence by minimizing direct resource competition. This is further supported by the varying degrees of dietary overlap among the species. For instance, the higher overlap between *A. vulgare* and *T. semiproiectus* (*O*_*jk*_ = 0.48) compared to *A. vulgare* and *S. raffaelei* (*O*_*jk*_ = 0.29) indicates that while some competition exists, it is mitigated by differences in food preferences and niche breadth (Uvarov and Goncharov 2023).

Interestingly, our results contrast with those of Bonato et al. (2021), who found that *Lithobius Validus* and *Eupolybothrus tridentinus* (both belonging to Lithobiomorpha) exhibited significantly higher dietary overlap with each other than with *Clinopodes flavidus* (Geophilomorpha). They attributed this pattern to phylogenetic effects and differences in functional traits related to feeding activity (Bortolin et al. 2018). In our study, however, *A. vulgare* and *S. raffaelei* , which are phylogenetically closer, displayed lower dietary overlap, suggesting that factors other than phylogenetic relatedness, such as fine‐scale resource partitioning or behavioral adaptations, may play a more significant role in shaping dietary preferences among terrestrial isopods.

*Trachelipus semiproiectus* , which belongs to a different family than the other two species, exhibited the broadest trophic niche width (*B* = 13.3987), consuming a diverse range of plant taxa, including Bryopsida and Polypodiopsida, which were absent in the diets of *A. vulgare* and *S. raffaelei* . This suggests that *T. semiproiectus* employs a generalist feeding strategy, allowing it to utilize a wider variety of plant resources and potentially reducing direct competition with the other two species. In contrast, *A. vulgare* and *S. raffaelei* , which are phylogenetically closer, displayed narrower niche widths (*B* = 6.0363 and *B* = 10.0396, respectively) and more specialized dietary composition, dominated by Sapindales and Rosales. This dietary specialization likely reflects their adaptation to specific resources, minimizing interspecific competition through niche partitioning (McCormack and Smith 2008; Carvalho and Cardoso 2020).

Multivariate ordination analyses (PCoA and NMDS) did not show significant clustering of species based on dietary composition, indicating continuous rather than discrete niche partitioning. This pattern suggests substantial dietary overlap at the community level, consistent with the generalist feeding strategies observed in terrestrial isopods (Saska 2008).

Our results revealed distinct differences in food niche width and interspecific overlap, supporting the niche differentiation theory as a key mechanism for species coexistence (Hardin 1960; Pianka 1973). *Trachelipus semiproiectus* exhibited the broadest trophic niche, consuming diverse plant taxa, including Bryopsida and Polypodiopsida, which were absent in the dietary composition of the other two species. In contrast, *A. vulgare* displayed a narrower niche, dominated by Sapindales, whereas *S. raffaelei* showed intermediate specialization with a preference for Rosales. These findings align with the hypothesis that species with overlapping distributions partition resources to minimize competition, thereby facilitating coexistence. The observed dietary divergence at taxonomic levels (order, family, genus) underscores the role of resource partitioning in structuring soil arthropod communities.

The dominance of Sapindales, Rosales, and Fabales in the diets of all three species highlights the importance of these plant orders as key food resources in the study area. However, the distinct preferences observed at the genus level (e.g., *A. vulgare* 's preference for *Koelreuteria* and *S. raffaelei* 's preference for *Broussonetia*) suggest that fine‐scale resource partitioning may play a critical role in reducing interspecific competition. This is consistent with previous studies emphasizing the role of dietary specialization in promoting species coexistence (Krebs 1999; Cooke and Crowley 2018).

In conclusion, this study highlights the importance of niche differentiation and dietary specialization in facilitating the coexistence of terrestrial isopod species. By leveraging advanced molecular techniques, we have gained new insights into the feeding ecology of these ecologically significant organisms. These findings not only contribute to our understanding of soil invertebrate communities but also provide a foundation for future research on biodiversity conservation and ecosystem management. To further our understanding of niche dynamics in terrestrial isopods, subsequent studies should: evaluate seasonal dietary variations to assess the temporal stability of niche partitioning, examine the physiological adaptations (such as digestive enzyme profiles or mandibular morphology) that underpin the observed dietary specializations, and investigate gut microbiome interactions to uncover microbial contributions to resource utilization. By integrating these methodologies, we can clarify the mechanistic drivers of coexistence within soil invertebrate communities.

## Author Contributions

**Jiachen Wang:** formal analysis (equal), methodology (lead), writing – original draft (lead). **Yiwen Yang:** resources (lead). **Gaoji Zhang:** formal analysis (equal), funding acquisition (lead). **Wei Xu:** writing – review and editing (equal). **Hongyi Liu:** conceptualization (lead), writing – review and editing (equal).

## Conflicts of Interest

The authors declare no conflicts of interest.

## Data Availability

DNA sequences in this study were deposited into the NCBI Sequence Read Archive (SRA) under accession number PRJNA1236731 (https://www.ncbi.nlm.nih.gov/).
